# Supplementary material for: DMRforPairs: identifying Differentially Methylated Regions between unique samples using array based methylation profiles
Source: BMC Bioinformatics. 2014 May 15;15:141. doi: 10.1186/1471-2105-15-141 (PMC4046028; doi:10.1186/1471-2105-15-141)
Supplement: Additional file 2 — DMRforPairs output for the comparison of A431-MCF7 and NA17018-NA17105. Please start from the HTML files in each folder. Available via the BMC Bioinformatics website. [file 1471-2105-15-141-S2.zip › 1394847754114233_MOESM2_ESM/A431_MCF7/figures/30095.pdf]

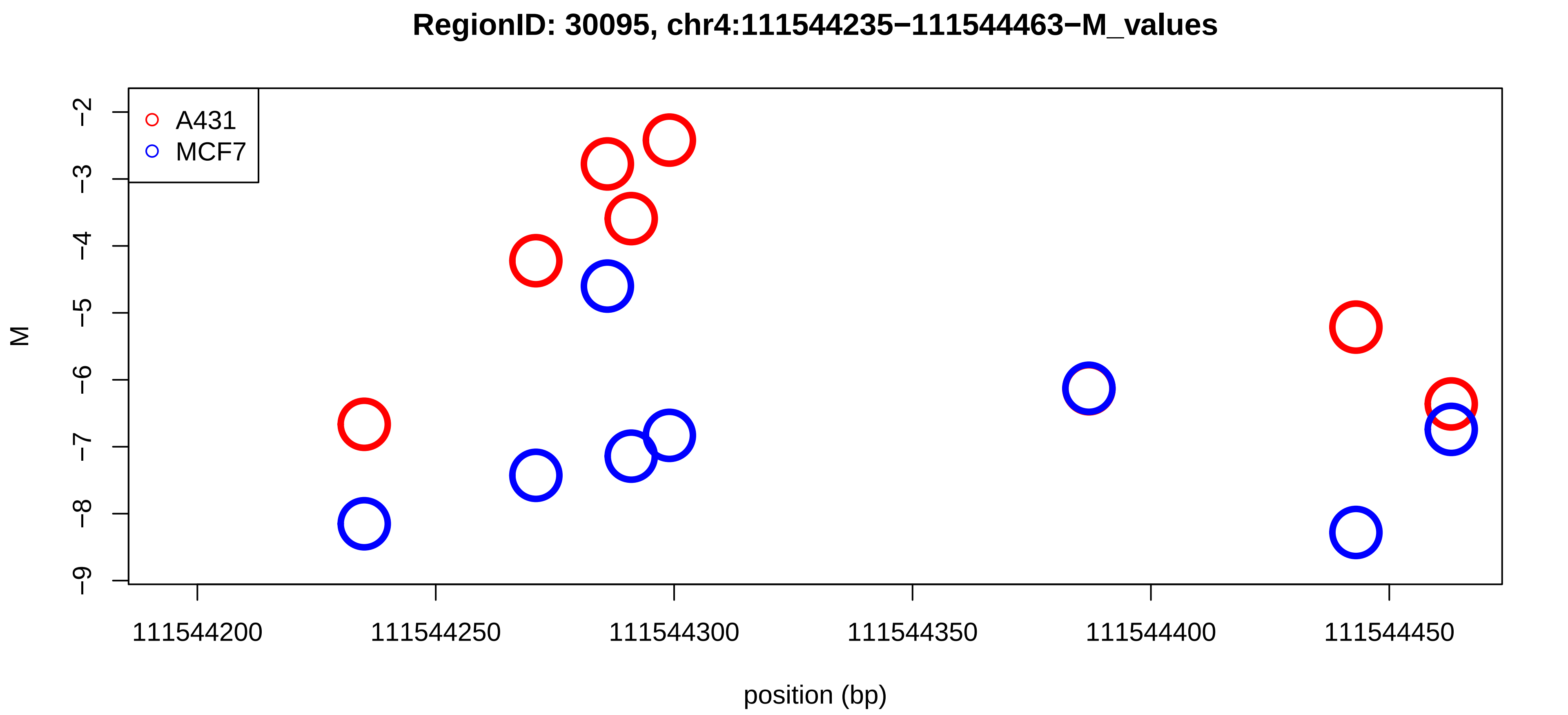
RegionID: 30095, chr4:111544235−111544463−M_values
A431
MCF7
M
−2
−3
−4
−5
−6
−7
−8
−9
111544200
111544250
111544300
111544350
111544400
111544450
position (bp)

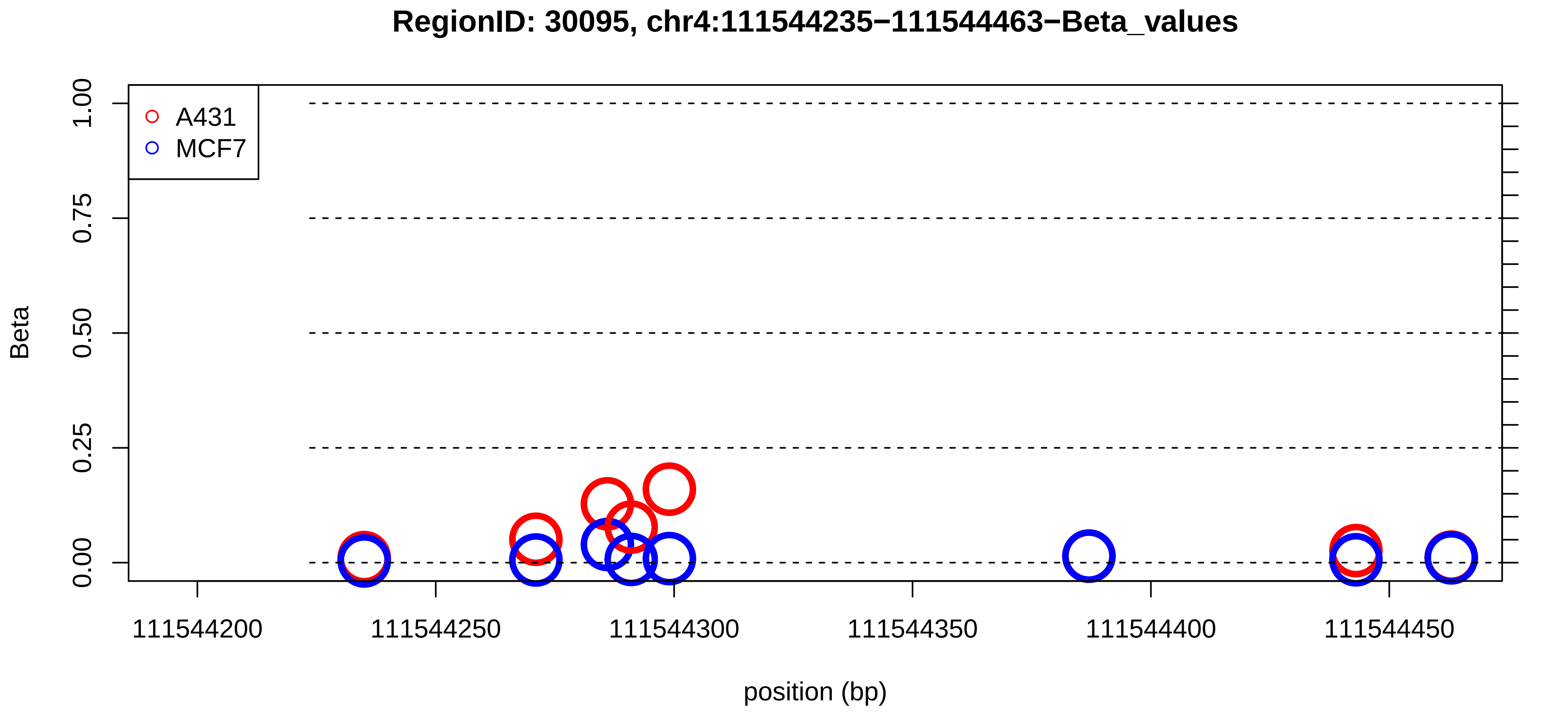
RegionID: 30095, chr4:111544235−111544463−Beta_values
A431
MCF7
Beta
1.00
0.75
0.50
0.25
0.00
111544200
111544250
111544300
111544350
111544400
111544450
position (bp)

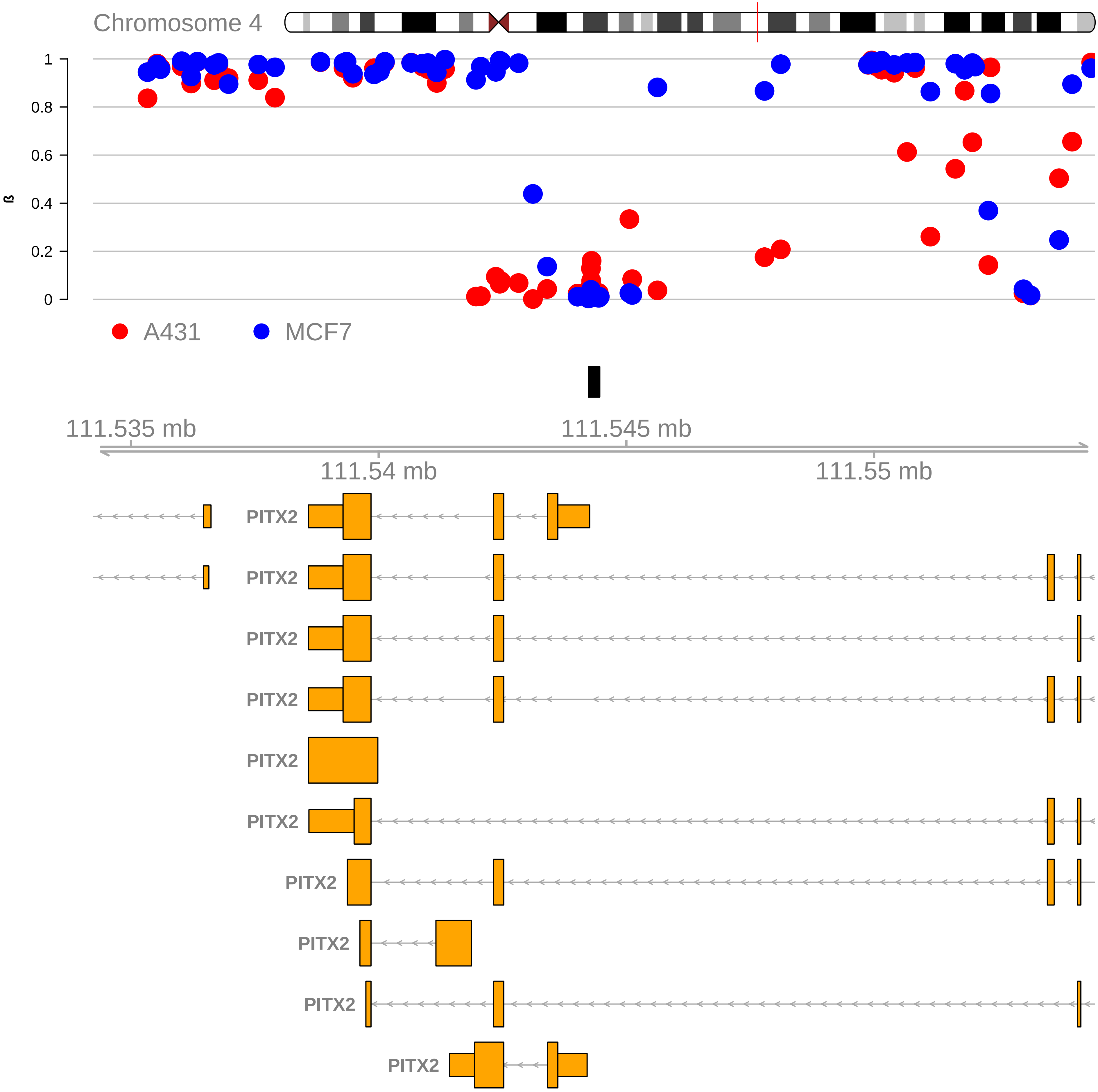
Chromosome 4
β
1
0.8
0.6
0.4
0.2
0
A431
MCF7
111.535 mb
111.545 mb
111.54 mb
111.55 mb
PITX2
PITX2
PITX2
PITX2
PITX2
PITX2
PITX2
PITX2
PITX2
PITX2
